# Efficient CRISPR/Cas9 Plasmids for Rapid and Versatile Genome Editing in *Drosophila*

**DOI:** 10.1534/g3.114.014126

**Published:** 2014-09-17

**Authors:** Joseph Gokcezade, Grzegorz Sienski, Peter Duchek

**Affiliations:** Institute of Molecular Biotechnology of the Austrian Academy of Sciences, 1030 Vienna, Austria

**Keywords:** *Drosophila*, CRISPR, Cas9, HDR

## Abstract

The CRISPR-associated RNA-guided nuclease Cas9 has emerged as a powerful tool for genome engineering in a variety of organisms. To achieve efficient gene targeting rates in *Drosophila*, current approaches require either injection of *in vitro* transcribed RNAs or injection into transgenic Cas9-expressing embryos. We report a simple and versatile alternative method for CRISPR-mediated genome editing in *Drosophila* using bicistronic Cas9/sgRNA expression vectors. Gene targeting with this single-plasmid injection approach is as efficient as in transgenic *nanos-Cas9* embryos and allows the isolation of targeted knock-out and knock-in alleles by molecular screening within 2 months. Our strategy is independent of genetic background and does not require prior establishment of transgenic flies.

The generation of targeted mutations and the precise modification of an organism’s genome sequence are powerful approaches to characterize the function of genes and regulatory elements. A prerequisite to achieve these goals efficiently is the induction of targeted double-strand breaks (DSBs) in the genome (Rouet *et al.* 1994). DSBs are typically repaired by error-prone nonhomologous end-joining (NHEJ), which often generates short insertions and deletions (indels), thereby causing frame-shift mutations (Bibikova *et al.* 2002). In the presence of a homologous donor, DSBs can also be repaired via homology-directed repair (HDR), a pathway that can be exploited to introduce specific nucleotide changes or generate defined insertions or deletions (Bibikova *et al.* 2003; Chen *et al.* 2011).

Of the various programmable nuclease platforms available for the generation of sequence-specific DSBs, the prokaryotic type II clustered regularly interspaced short palindromic repeat (CRISPR) adaptive immune system has attracted considerable attention for its versatility and ease of use (Sander and Joung 2014; Hsu *et al.* 2014). The CRISPR-associated Cas9 endonuclease can recognize and cleave target DNA in combination with a single chimeric guide RNA (sgRNA), where the first 20 nucleotides of the RNA provide sequence-specificity by guiding Cas9 to complementary DNA molecules (Jinek *et al.* 2012; Gasiunas *et al.* 2012). By replacing these 20 nucleotides of the sgRNA, this simple two-component system can be readily reprogrammed to target a sequence of choice, making it ideally suited for genome engineering purposes (Jinek *et al.* 2012).

Although the molecular details of the CRISPR/Cas9 mechanism were elucidated only recently, this RNA-guided nuclease (RGN) has already been applied to edit the genomes in a wide variety of organisms (Sander and Joung 2014; Hsu *et al.* 2014). Previous attempts of Cas9-mediated gene targeting in *Drosophila* initially achieved low mutation rates (Gratz *et al.* 2013) and required *in vitro* transcription of *Cas9* and the *sgRNA* (Yu *et al.* 2013; Bassett *et al.* 2013; Yu *et al.* 2014) or injecting into transgenic *Cas9* expressing embryos (Kondo and Ueda 2013; Ren *et al.* 2013; Sebo *et al.* 2014; Gratz *et al.* 2014; Port *et al.* 2014) to achieve feasible targeting rates.

We describe a highly efficient, fast, and versatile method to generate targeted mutations and perform precise genome engineering in less than 2 months. Our strategy is independent of genetic background and based on injecting a bicistronic plasmid that contains both components required to induce targeted DSBs, *Cas9* and the *sgRNA*, under the control of constitutive *Drosophila* promoters.

## Materials and Methods

### Generation of pDCC plasmids

**Figure 1:**
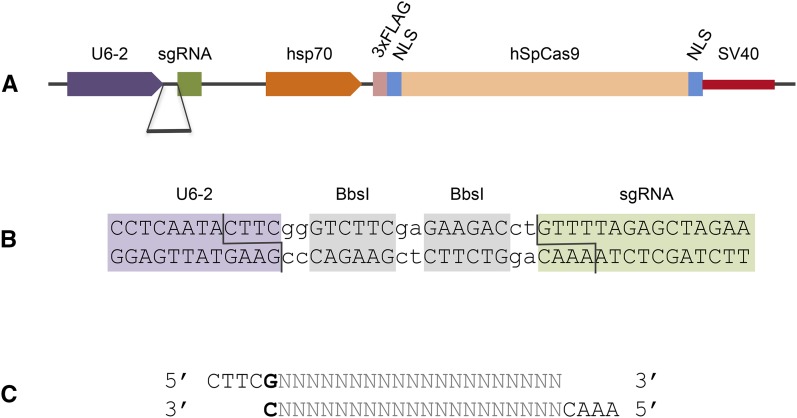
A bicistronic *Drosophila* CRISPR/Cas9 vector. (A) Schematic map of pDCC6, with the sgRNA cassette under the control of the *Drosophila U6:96Ab* (U6-2) promoter as well as an *hsp70Bb* promoter driving *Cas9* expression. (B) gRNA sequences are inserted between the U6 promoter and the sgRNA scaffold via two *Bbs*I sites. (C) gRNAs are cloned as complementary oligonucleotide pairs with suitable overhangs and an additional G (bold, required for RNA PolIII transcription) preceding the target-specific 20 nt protospacer (gray).

The human codon-optimized *Streptococcus pyogenes Cas9*^D10A^ nickase (*hSpCas9D10A*) was cloned from pX335 (Cong *et al.* 2013) into the pHW *Drosophila* Gateway vector and the *Bbs*I site in the SV40 3′-UTR mutated by PCR. The sgRNA expression cassette was then inserted into the *Not*I site of pHW-Cas9D10A by In-Fusion cloning (Clontech) of the *U6:96Ab* promoter amplified from genomic DNA and the chimeric sgRNA scaffold from pX335, resulting in plasmid pDCC1. To generate pDCC2, the N-terminus of *Cas9*^D10A^ in pDCC1 was replaced with an *Age*I-*Apa*I fragment of wild-type *Cas9* from pX330 (Cong *et al.* 2013). Finally, we replaced the *hsp70Ab* promoter between the *Xba*I and *Hin*dIII sites in pDCC1 and pDCC2 with a 350-bp *hsp70Bb* promoter fragment to obtain plasmids pDCC5 and pDCC6, respectively (Figure 1A and Supporting Information, Figure S1).

### gRNA cloning and embryo injections

Genomic regions downstream of the start codon (for NHEJ mutagenesis) or close to the stop codon (for HDR) were sequenced in the injection stocks to avoid SNPs. The sequences were then scanned for NGG protospacer adjacent motifs (PAM) and suitable gRNAs were checked for potential off-targets with the CRISPR Optimal Target Finder (Gratz *et al.* 2014).

All gRNAs were preceded by a 5′-G required for RNA Pol III transcription from the U6 promoter and cloned as complementary oligonucleotides with suitable overhangs for the *Bbs*I site (Figure 1). The oligos were phosphorylated with polynucleotide kinase (Thermo Scientific), annealed, and ligated into *Bbs*I-digested and dephosphorylated pDCC6 or pU6-BbsI-chiRNA vectors. Sequence-verified plasmid DNA (Midi-Prep, Qiagen) was injected into dechorionated embryos at 100 ng/µl as described (Ringrose 2009). For HDR, ultramer DNA oligos (IDT) were co-injected at 150 ng/µl (*ham*) or 250 ng/µl (*dpn*, *mγ*).

### Fly crosses

pDCC6-e was injected into isogenized *w*^1118^ embryos, and pU6-e was injected into *nanos-Cas9* (Port *et al.* 2014) and *vasa-Cas9* (Gratz *et al.* 2014). Hatched G0 flies were crossed to TM3, *Sb*, *e*/TM6, *Hu*, *Tb*, *e*, and dark flies were counted in the next generation. pDCC6-y and pDCC6-w were co-injected into Canton-S embryos and subsequently crossed to *y*, *w* mutant flies. When targeting *ebony* as well as *yellow* and *white*, two rounds of injections were performed on different days, and the results from both injections were combined (Table 1 and Table 2).

**Table 1:** Germline transmission rates for NHEJ-mediated mutagenesis and ssODN-mediated HDR

				Male Crosses		Female crosses		Total
Construct	Host	ssODN	Embryos	% (No.) Founders	% (No.) Progeny	% (No.) Founders	% (No.) Progeny	% F1
DCC6-e	w^1118^	—	189	89 (17/19)	14 (386/2802)	57 (26/46)	12 (536/4312)	13
U6-e	nos-cas9	—	192	53 (9/17)	5.9 (68/1143)	64 (9/14)	16 (153/954)	11
U6-e	vas-cas9	—	201	83 (10/12)	29 (362/1270)	94 (32/34)	49 (1765/3572)	44
DCC6-MED27	FRT82	—	75	43 (3/7)	20 (7/35)	0 (0/5)	0 (0/25)	12
DCC6-ham	w^1118^	V5	85	14 (3/22)	2.7 (3/110)	6.7 (1/15)	6.7 (4/60)	4.1
DCC6-dpn	w^1118^	V5	126	11 (1/9)	2.8 (2/72)	13 (2/15)	2.5 (3/120)	2.6
DCC6-mγ	w^1118^	V5	191	15 (3/20)	11 (11/100)	5.9 (1/17)	5.9 (5/85)	8.6

**Table 2:** Mutation rates for simultaneously targeting *yellow* and *white*

			G0 Founders			F1 Mutants		
Construct	Host	Embryos	y^−^	w^−^	y^−^ w^−^	y^−^ w^+^	y^+^ w^−^	y^−^ w^−^
DCC6-y + DCC6-w	Canton-S	167	31/35	26/35	20/35	23%	4.7%	15%

pDCC6-MED27 was injected into *FRT82B* embryos and G0 flies crossed to *Ly*/TM3, *Sb*. From each fertile G0 cross, five *Ly* males were individually crossed to Df*(Exel7283)*/TM3, *Sb* virgins.

For HDR, pDCC6 plasmids and donor oligos were co-injected into an isogenized *w*^1118^ stock and hatched G0 flies crossed to balancer flies appropriate for the target chromosome. Between four and eight males from each fertile cross were individually crossed to balancer virgins again. In the next generation, males were collected for PCR.

### PCR screening

Flies were homogenized in a 96-well format (1–3 flies per well) together with 100 µl squishing buffer (Gloor *et al.* 1993) and a 5-mm steel bead (Qiagen) by vigorous shaking in a TissueLyser II (Qiagen) for 10 min at 28 Hz. The plates were then incubated at 65° for 2 hr, followed by an inactivation step at 94° for 2 min; 1 µl of the supernatant was used for PCR (25 µl volume, 35 cycles). For HDR screening, undiluted PCR samples were analyzed with a high-resolution 96-capillary fragment analyzer (Advanced Analytical).

Prior to sequencing, 6 µl of each PCR were enzymatically purified with 0.5 µl of Illustra ExoProStar 1-Step enzyme mix (GE Healthcare) at 37° for 30 min, followed by inactivation at 80° for 20 min.

## Results

### An efficient CRISPR vector for *Drosophila*

To adapt the CRISPR/Cas9 system for gene targeting in *Drosophila*, we generated the plasmid pDCC6 (Figure 1), which contains the *Cas9* gene from *S. pyogenes* under the control of the *hsp70Bb* promoter (Gratz *et al.* 2013) and a *sgRNA* cassette downstream of the *U6:96Ab* promoter (Gratz *et al.* 2013; Kondo and Ueda 2013; Ren *et al.* 2013; Xue *et al.* 2014).

As an initial test, we targeted the *ebony* (*e*) gene on the third chromosome, which when mutated leads to a darker pigmentation of the adult cuticle that is simple to score. A gRNA target site was identified just downstream of the start codon (Port *et al.* 2014) and cloned into pDCC6 as a pair of oligonucleotides. Flies injected with pDCC6-e were crossed to homozygous *e^1^* mutants and the number of dark offspring were counted in the next generation. From 65 fertile G0 flies, 43 gave rise to *ebony* mutants (66% founders), with 13% of all F1 flies being *ebony* (Table 1). Sequencing of genomic DNA from randomly chosen *ebony* flies revealed indels leading to frame-shifts close to the gRNA target site (File S1), characteristic of DSB repair by NHEJ.

### Comparison with transgenic *Cas9* flies

Several laboratories have recently generated transgenic flies that express *Cas9* under the control of either germline-specific or ubiquitous promoters (Kondo and Ueda 2013; Sebo *et al.* 2014; Ren *et al.* 2013; Gratz *et al.* 2014; Port *et al.* 2014). To compare the targeting rates of the bicistronic pDCC6 plasmid with sgRNA plasmid injections into published transgenic *Cas9* flies, we cloned the same gRNA that targets *ebony* into pU6-BbsI-chiRNA (Gratz *et al.* 2013). This vector also makes use of the *U6:96Ab* promoter to drive *sgRNA* expression but lacks the *Cas9* gene. The resulting pU6-e plasmid was injected into *nanos-Cas9* (Port *et al.* 2014) and *vasa-Cas9* (Gratz *et al.* 2014) expressing embryos. After crossing G0 flies to *e*^1^ mutants, 11% from the *nanos-Cas9* injections had a dark body color, a slightly lower frequency than what we observed after pDCC6-e injection into *w^1118^* embryos (13%). In contrast, injection of *vasa-Cas9* flies resulted in a mutation rate of 44%, a 3.4-fold increase over our bicistronic vector (Table 1).

### Mutagenesis of an essential gene

Using the bicistronic pDCC6 vector, we next aimed to generate mutations in an essential gene whose loss does not result in a visible phenotype and chose to target subunit 27 of the mediator complex (*MED27*). Because *MED27* is located at cytological position 83B, pDCC6-MED27 was injected into *FRT82B* embryos, which would subsequently allow the generation of mitotic clones without having to recombine the mutant alleles onto the FRT chromosome.

Five males from each of the 12 fertile G0 crosses were individually mated to deficiency virgins uncovering the *MED27* locus. Of the 60 crosses analyzed, seven did not complement the deficiency (11.7%) (Table 1). Sequencing of the gRNA target site in these flies revealed indels leading to frame-shifts in the *MED27* coding sequence (File S1). It is thus feasible to isolate loss-of-function alleles of an essential gene at frequencies high enough for fast screening in the absence of a visible phenotype and in a genetic background of choice.

### Generation of double mutants

The high mutation rates achieved when targeting single genes led us to investigate whether we could mutate two genes simultaneously. For this purpose, a mix of two pDCC6 plasmids that target the body color gene *yellow* (*y*) and the eye color gene *white* (*w*) on the X chromosome were co-injected into wild-type embryos. Screening F1 flies, we observed targeting rates of 38% and 20% for *yellow* and *white*, respectively, and 15% of all offspring were phenotypically both *yellow* and *white* (Table 2). Thus, it is possible to multiplex our vector to generate double mutants with high efficiency in a wild-type background.

### HDR with oligonucleotide donors

The use of single-stranded oligodeoxynucleotides (ssODN) as HDR donors is a simple, yet powerful, approach to introduce precise changes in the genome (Chen *et al.* 2011). We set out to add a V5 epitope tag to the C-terminus of the transcription factors Hamlet (Ham), Deadpan (Dpn), and E(spl)mγ-HLH (mγ) with oligos containing the 42 nucleotide V5 sequence, flanked by homology arms ranging from 61 to 71 nucleotides in length. Each ssODN was co-injected into *w^1118^* embryos with a pDCC6 plasmid that targets the corresponding gene close to the stop codon (File S1).

Using PCR primers distal to the homology arms, the genomic region surrounding the target site was amplified from heterozygous F2 flies. High-resolution capillary gel electrophoresis identified 7/170 (*ham*), 5/190 (*dpn*), and 16/185 (*mγ*) that had amplified a fragment of wild-type length as well as an additional fragment approximately 45 bp larger, indicative of V5 integration via HDR (Table 1, Figure S2). For 3/7 (*ham*), 5/5 (*dpn*), and 6/16 (*mγ*) lines, the integrity of the tag could be confirmed by DNA sequencing. The remaining lines lacked one or two nucleotides within or close to the V5 sequence, probably due to shorter donor molecules present in the injection mix, which is common for synthesized oligos of that length.

Using our bicistronic vector, we could demonstrate that it is feasible to identify HDR-mediated targeting events in the absence of a visible marker by PCR screening in a standard genetic background that is independent of transgenic *Cas9* expression.

## Discussion

Whereas previous reports of using CRISPR/Cas9 in *Drosophila* required the injection of *in vitro* transcribed RNA or injecting into transgenic *Cas9*-expressing embryos to achieve feasible gene targeting rates (Yu *et al.* 2013; Bassett *et al.* 2013; Kondo and Ueda 2013; Sebo *et al.* 2014; Ren *et al.* 2013; Gratz *et al.* 2014; Yu *et al.* 2014; Port *et al.* 2014), we chose a plasmid-based approach and reasoned that having both *Cas9* and the *sgRNA* on a single vector might enhance targeting efficiencies (Cong *et al.* 2013; Wang *et al.* 2013). We obtained germline transmission rates of >10% for mutations in *ebony* and the essential gene *MED27*, as well as for simultaneously targeting *yellow* and *white*. Using three ssODN donors, we identified HDR events in 2.6–8.6% of F1 flies, similar to frequencies reported with a dsRed-marked plasmid donor injected into *vasa*-*Cas9* embryos (Gratz *et al.* 2014).

Although mutations in *MED27* were isolated by screening for lethality, detecting mutant alleles by phenotype is not feasible for every gene. Because mutation rates are usually above 10% following pDCC6 injection, we now routinely sequence PCR products amplified from 50 to 100 heterozygous F2 flies to identify those with frame-shift mutations, or from 200 flies for ssODN-mediated HDR.

Transgenic flies that constitutively express Cas9 have recently been used to increase targeting frequencies (Kondo and Ueda 2013; Ren *et al.* 2013; Sebo *et al.* 2014; Gratz *et al.* 2014; Port *et al.* 2014). However, we did not observe higher mutation rates when targeting *ebony* in *nanos-Cas9* flies over our single-plasmid injection approach, but we obtained a 3.4-fold increase with *vasa-Cas9* embryos. For many applications, the ability to induce mutations in a genetic background of choice will outweigh the increased targeting rates in *vasa-Cas9* flies. In addition, *vasa-Cas9* expression is not restricted to the germline (Port *et al.* 2014), and sgRNA injected *vasa*-*Cas9* embryos were reported to have low viability and fertility rates, especially when targeting essential genes (Sebo *et al.* 2014; Port *et al.* 2014). In contrast, we did not notice patches of mutant tissue in adult G0 flies when targeting *ebony* or *yellow*, indicating that mutations in somatic cells after pDCC6 injection are rare, despite efficient targeting in the germline. Another advantage of our bicistronic vectors is that they are independent of host genotypes, which we have exploited to mutate genes on FRT chromosomes. For such cases, it would be time-consuming and laborious either to first cross in a *Cas9* transgene or to recombine the mutant alleles afterward.

Co-injecting two pDCC6 plasmids that express different sgRNAs, we were able to simultaneously mutate two genes on the same chromosome. A similar strategy could also be applied to generate large deletions, for which two DSBs are required. In conjunction with the Cas9 nickase (*Cas9*^D10A^ present in pDCC5), this enables a double-nicking/offset-nicking approach, which has been shown to reduce the indel frequency at off-target sites in mammalian cells (Mali *et al.* 2013; Ran *et al.* 2013).

In summary, we describe versatile tools for Cas9-mediated genome editing in *Drosophila* that are independent of genetic background, yet efficient enough for PCR-based screening to be feasible. For each targeting experiment, one simply needs to clone a pair of oligonucleotides into the targeting vector prior to injection, without the need for *in vitro* transcription reactions, thus making genome engineering as straightforward as regular transgenesis. Finally, by exchanging the promoters that drive *Cas9* and the *sgRNA*, the single-plasmid approach could be adapted for genome engineering in other species that are amenable to DNA injections.

## Supplementary Material

Supporting Information
