# Supplementary material for: Efficient CRISPR/Cas9 Plasmids for Rapid and Versatile Genome Editing in Drosophila
Source: G3 (Bethesda). 2014 Sep 17;4(11):2279–82. doi: 10.1534/g3.114.014126 (PMC4232553; doi:10.1534/g3.114.014126)
Supplement: Supporting Information [file supp_4_11_2279__index.html]

Efficient CRISPR/Cas9 Plasmids for Rapid and Versatile Genome Editing in Drosophila — Supporting Information 

# Efficient CRISPR/Cas9 Plasmids for Rapid and Versatile Genome Editing in *Drosophila*

## Supporting Information for Gokcezade, Sienski, and Duchek, 2014

**Files in this Data Supplement:**

- Supporting Information - File S1 and Figures S1-S2 (PDF, 604 KB)
- File S1 - Supplementary Information (PDF, 141 KB)
- Figure S1 - Nucleotide sequence of pDCC6. (PDF, 114 KB)
- Figure S2 - Detection of HDR-mediated V5-tag integration by high-resolution capillary electrophoresis. (PDF, 365 KB)
