# Supplementary material for: Efficient CRISPR/Cas9 Plasmids for Rapid and Versatile Genome Editing in Drosophila
Source: G3 (Bethesda). 2014 Sep 17;4(11):2279–82. doi: 10.1534/g3.114.014126 (PMC4232553; doi:10.1534/g3.114.014126)
Supplement: Supporting Information [file supp_g3.114.014126_014126SI.pdf]

G3·Genes | Genomes | Genetics

Efficient CRISPR/Cas9 Plasmids for Rapid and Versatile Genome Editing in *Drosophila*

Joseph Gokcezade, Grzegorz Sienski, and Peter Duchek

Institute of Molecular Biotechnology of the Austrian Academy of Sciences (IMBA), 1030 Vienna, Austria

Corresponding Author

Peter Duchek

IMBA

Dr Bohrgasse 3

1030 Vienna

Austria

Phone: +43-1-79044 4509

Email: peter.duchek@imba.oeaw.ac.at

DOI: 10.1534/g3.114.014126

File S1

Supplementary Information

gRNA sequences

e	GCCACAATTGTCGATCGTCA	(Port *et al.* 2014)
y	GCGATATAGTTGGAGCCAGC	(Gratz *et al.* 2014)
w	CCGAGAACCTCACCTATGCC	
MED27	CTCCTCGCCGCTCTCCCCAT	
ham	GGCCATTACAATAAGCTCTT	
dpn	CCTGTGCGGTTGATCTACCA	
mγ	GTGAAGTAATCCTATCTACC	

Sequences of recovered indel alleles

e

ATGGGTTCGCT**GCCACAATTGTCGATCGTCA**AGGGTCTGCAGCAAGACTT wt
ATGGGTTCGCT**GCCACAATTGTCGATC-TCA**AGGGTCTGCAGCAAGACTT - 1
ATGGGTTCGCT**GCCACAATTGTC**--------------TGCAGCAAGACTT -14
ATGGGTTCGCT**GCCACAATTGTCGA**----**CA**AGGGTCTGCAGCAAGACTT - 4
ATGGGTTCGCT**GCCACAATTGT**-----**GTCA**AGGGTCTGCAGCAAGACTT - 5
ATGGGTTCGCT**GCCA**----------------AGGGTCTGCAGCAAGACTT -16
ATGGGTTCGCT**GCCAC**--------------**A**AGGGTCTGCAGCAAGACTT -14
ATGGGTTCGCT**GCCACAATTGTCGATC**----------TGCAGCAAGACTT -10
ATGGGTTCGCT**GCCACAATTGTCGATC**-tgcaaga-CTGCAGCAAGACTT - 9/+7
ATGGGTTCGCT**GCCACAATTGTCGATCG**a**TCA**AGGGTCTGCAGCAAGACTT + 1

MED27

GCCGATGGAACCG**ATGGGGAGAGCGGCGAGGAG**AGCCG wt
GCCGATGGAACCG**ATG**ctcgaacc**GAGCGGCGAGGAG**AGCCG -4/+8
GCCGATGGAACCG**ATG-GGAGAGCGGCGAGGAG**AGCCG -1
GCCGATGGAACCG**ATG**ggagagcggagat**GGGAGAGCGGCGAGGAG**AGCCG +13

ssODN donor sequences

The homology arms are in upper case, the central V5 sequence in lower case, and the stop codons underlined. The gRNA target sequences are in bold, and are followed by a PAM (NGG). Silent nucleotide changes were introduced in the standard V5 sequence to create a *Hin*dIII restriction site. Donor sequences were synthesized in the same orientation as the gRNA. For all cases presented here, the insertion of the V5 tag destroyed the gRNA target site so that V5-tagged chromosomes would not be recognized and cleaved again.

ham-V5

GCAACATCAGCAACAACAACAGCAGCAGCGGCAACAACAACAACAGCTCCAA**GGCCATTACA**
ATAAGCTCTggtaagcctatacctaaccctcttcttggtctagatagcacg**T**AGGAATCGTA
AGAGATTAGAGAGCTTGCTTTCCTCGTTGTTAAATGATAAACTTACTTTAACTC

dpn-V5

GCAATGAAATAAATTAACAATGTATAAACGTAACATAGAAGTGG**CCTGTGCGGTTGATCTA**c
gtgctatctagaccaagaagagggttaggtataggcttacc**CCA**CGGCCTCCAAGCGGAGCT
CGACTTTTCGATGGCGCCAGCTAGGAATTTCTTGGACGAGGGC

mγ-V5

CTTTCTATGATCTCTCCTTCGTTTTTTTTTTTTTCAGTTGTGGT**GTGAAGTAATCCTATCTA**
cgtgctatctagaccaagaagagggttaggtataggcttacc**CC**AGGGACGCCAGACGTTCT
CCTCGTCCTCGCTGTCGTCCTCCATCTTGGTCACATCGATGGTGGTC

GCGCCCAATACGCAAACCGCCTCTCCCCGCGCGTTGGCCGATTCATTAATGCAGGCAACTCGTGAAAGGTAGGCG
GATCAGCGGTTCGACTTGCAGCCTGAAATACGGCACGAGTAGGAAAAGCCGAGTCAAATGCCGAATGCAGAGTCT
CATTACAGCACAATCAACTCAAGAAAAACTCGACACTTTTTTACCATTTGCACTTAAATCCTTTTTTATTCGTTA
TGTATACTTTTTTTGGTCCCTAACCAAAACAAAACCAAACTCTCTTAGTCGTGCCTCTATATTTAAAACTATCAA
TTTATTATAGTCAATAAATCGAACTGTGTTTTCAACAAACGAACAATAGGACACTTTGATTCTAAAGGAAATTTT
GAAAATCTTAAGCAGAGGGTTCTTAAGACCATTTGCCAATTCTTATAATTCTCAACTGCTCTTTCCTGATGTTGA
TCATTTATATAGGTATGTTTTCCTCAATACTTCggGTCTTCgaGAAGACctGTTTTAGAGCTAGAAATAGCAAGT
TAAAATAAGGCTAGTCCGTTATCAACTTGAAAAAGTGGCACCGAGTCGGTGCTTTTTTGTTTTAGAGCTAGAAAT
AGCAAGTTAAAATAAGGCTAGTCCGTTTTTAGCGCGTGCGCCAATTCTGCAGACAAATGGCTCTAGAATCCCAAA
ACAAACTGGTTATTGTGGTAGGTCATTTGTTTGGCAGAAAGAAAACTCGAGAAATTTCTCTGGCCGTTATTCGTT
ATTCTCTCTTTTCTTTTTGGGTCTCTCCCTCTCTGCACTAATGCTCTCTCACTCTGTCACACAGTAAACGGCATA
CTGCTCTCGTTGGTTCGAGAGAGCGCGCCTCGAATGTTCGCGAAAAGAGCGCCGGAGTATAAATAGAGGCGCTTC
GTCTACGGAGCGACAATTCAATTCAAACAAGCAAAGTGAACACGTCGCTAAGCGAAAGCTAAGCAAATAAACAAG
CGCAGCTGAACAAGCTAAACAATCTGCAGCCAAGCTCGATAAGCTTGTTCGAATCTCGAGTGCGCGCTTCCGGAG
GTATACACCTAGGCGGTACCACTGCAGTGAATTCGGAGCTCTACCGGTGCCACCATGGACTATAAGGACCACGAC
GGAGACTACAAGGATCATGATATTGATTACAAAGACGATGACGATAAGATGGCCCCAAAGAAGAAGCGGAAGGTC
GGTATCCACGGAGTCCCAGCAGCCGACAAGAAGTACAGCATCGGCCTGGACATCGGCACCAACTCTGTGGGCTGG
GCCGTGATCACCGACGAGTACAAGGTGCCCAGCAAGAAATTCAAGGTGCTGGGCAACACCGACCGGCACAGCATC
AAGAAGAACCTGATCGGAGCCCTGCTGTTCGACAGCGGCGAAACAGCCGAGGCCACCCGGCTGAAGAGAACCGCC
AGAAGAAGATACACCAGACGGAAGAACCGGATCTGCTATCTGCAAGAGATCTTCAGCAACGAGATGGCCAAGGTG
GACGACAGCTTCTTCCACAGACTGGAAGAGTCCTTCCTGGTGGAAGAGGATAAGAAGCACGAGCGGCACCCCATC
TTCGGCAACATCGTGGACGAGGTGGCCTACCACGAGAAGTACCCCACCATCTACCACCTGAGAAAGAAACTGGTG
GACAGCACCGACAAGGCCGACCTGCGGCTGATCTATCTGGCCCTGGCCCACATGATCAAGTTCCGGGGCCACTTC
CTGATCGAGGGCGACCTGAACCCCGACAACAGCGACGTGGACAAGCTGTTCATCCAGCTGGTGCAGACCTACAAC
CAGCTGTTCGAGGAAAACCCCATCAACGCCAGCGGCGTGGACGCCAAGGCCATCCTGTCTGCCAGACTGAGCAAG
AGCAGACGGCTGGAAAATCTGATCGCCCAGCTGCCCGGCGAGAAGAAGAATGGCCTGTTCGGAAACCTGATTGCC
CTGAGCCTGGGCCTGACCCCCAACTTCAAGAGCAACTTCGACCTGGCCGAGGATGCCAAACTGCAGCTGAGCAAG
GACACCTACGACGACGACCTGGACAACCTGCTGGCCCAGATCGGCGACCAGTACGCCGACCTGTTTCTGGCCGCC
AAGAACCTGTCCGACGCCATCCTGCTGAGCGACATCCTGAGAGTGAACACCGAGATCACCAAGGCCCCCCTGAGC
GCCTCTATGATCAAGAGATACGACGAGCACCACCAGGACCTGACCCTGCTGAAAGCTCTCGTGCGGCAGCAGCTG
CCTGAGAAGTACAAAGAGATTTTCTTCGACCAGAGCAAGAACGGCTACGCCGGCTACATTGACGGCGGAGCCAGC
CAGGAAGAGTTCTACAAGTTCATCAAGCCCATCCTGGAAAAGATGGACGGCACCGAGGAACTGCTCGTGAAGCTG
AACAGAGAGGACCTGCTGCGGAAGCAGCGGACCTTCGACAACGGCAGCATCCCCCACCAGATCCACCTGGGAGAG
CTGCACGCCATTCTGCGGCGGCAGGAAGATTTTTACCCATTCCTGAAGGACAACCGGGAAAAGATCGAGAAGATC
CTGACCTTCCGCATCCCCTACTACGTGGGCCCTCTGGCCAGGGGAAACAGCAGATTCGCCTGGATGACCAGAAAG
AGCGAGGAAACCATCACCCCCTGGAACTTCGAGGAAGTGGTGGACAAGGGCGCTTCCGCCCAGAGCTTCATCGAG
CGGATGACCAACTTCGATAAGAACCTGCCCAACGAGAAGGTGCTGCCCAAGCACAGCCTGCTGTACGAGTACTTC
ACCGTGTATAACGAGCTGACCAAAGTGAAATACGTGACCGAGGGAATGAGAAAGCCCGCCTTCCTGAGCGGCGAG
CAGAAAAAGGCCATCGTGGACCTGCTGTTCAAGACCAACCGGAAAGTGACCGTGAAGCAGCTGAAAGAGGACTAC
TTCAAGAAAATCGAGTGCTTCGACTCCGTGGAAATCTCCGGCGTGGAAGATCGGTTCAACGCCTCCCTGGGCACA
TACCACGATCTGCTGAAAATTATCAAGGACAAGGACTTCCTGGACAATGAGGAAAACGAGGACATTCTGGAAGAT
ATCGTGCTGACCCTGACACTGTTTGAGGACAGAGAGATGATCGAGGAACGGCTGAAAACCTATGCCCACCTGTTC
GACGACAAAGTGATGAAGCAGCTGAAGCGGCGGAGATACACCGGCTGGGGCAGGCTGAGCCGGAAGCTGATCAAC
GGCATCCGGGACAAGCAGTCCGGCAAGACAATCCTGGATTTCCTGAAGTCCGACGGCTTCGCCAACAGAAACTTC
ATGCAGCTGATCCACGACGACAGCCTGACCTTTAAAGAGGACATCCAGAAAGCCCAGGTGTCCGGCCAGGGCGAT
AGCCTGCACGAGCACATTGCCAATCTGGCCGGCAGCCCCGCCATTAAGAAGGGCATCCTGCAGACAGTGAAGGTG
GTGGACGAGCTCGTGAAAGTGATGGGCCGGCACAAGCCCGAGAACATCGTGATCGAAATGGCCAGAGAGAACCAG
ACCACCCAGAAGGGACAGAAGAACAGCCGCGAGAGAATGAAGCGGATCGAAGAGGGCATCAAAGAGCTGGGCAGC
CAGATCCTGAAAGAACACCCCGTGGAAAACACCCAGCTGCAGAACGAGAAGCTGTACCTGTACTACCTGCAGAAT
GGGCGGGATATGTACGTGGACCAGGAACTGGACATCAACCGGCTGTCCGACTACGATGTGGACCATATCGTGCCT
CAGAGCTTTCTGAAGGACGACTCCATCGACAACAAGGTGCTGACCAGAAGCGACAAGAACCGGGGCAAGAGCGAC
AACGTGCCCTCCGAAGAGGTCGTGAAGAAGATGAAGAACTACTGGCGGCAGCTGCTGAACGCCAAGCTGATTACC
CAGAGAAAGTTCGACAATCTGACCAAGGCCGAGAGAGGCGGCCTGAGCGAACTGGATAAGGCCGGCTTCATCAAG
AGACAGCTGGTGGAAACCCGGCAGATCACAAAGCACGTGGCACAGATCCTGGACTCCCGGATGAACACTAAGTAC
GACGAGAATGACAAGCTGATCCGGGAAGTGAAAGTGATCACCCTGAAGTCCAAGCTGGTGTCCGATTTCCGGAAG
GATTTCCAGTTTTACAAAGTGCGCGAGATCAACAACTACCACCACGCCCACGACGCCTACCTGAACGCCGTCGTG
GGAACCGCCCTGATCAAAAAGTACCCTAAGCTGGAAAGCGAGTTCGTGTACGGCGACTACAAGGTGTACGACGTG
CGGAAGATGATCGCCAAGAGCGAGCAGGAAATCGGCAAGGCTACCGCCAAGTACTTCTTCTACAGCAACATCATG
AACTTTTTCAAGACCGAGATTACCCTGGCCAACGGCGAGATCCGGAAGCGGCCTCTGATCGAGACAAACGGCGAA
ACCGGGGAGATCGTGTGGGATAAGGGCCGGGATTTTGCCACCGTGCGGAAAGTGCTGAGCATGCCCCAAGTGAAT
ATCGTGAAAAAGACCGAGGTGCAGACAGGCGGCTTCAGCAAAGAGTCTATCCTGCCCAAGAGGAACAGCGATAAG
CTGATCGCCAGAAAGAAGGACTGGGACCCTAAGAAGTACGGCGGCTTCGACAGCCCCACCGTGGCCTATTCTGTG
CTGGTGGTGGCCAAAGTGGAAAAGGGCAAGTCCAAGAAACTGAAGAGTGTGAAAGAGCTGCTGGGGATCACCATC
ATGGAAAGAAGCAGCTTCGAGAAGAATCCCATCGACTTTCTGGAAGCCAAGGGCTACAAAGAAGTGAAAAAGGAC
CTGATCATCAAGCTGCCTAAGTACTCCCTGTTCGAGCTGGAAAACGGCCGGAAGAGAATGCTGGCCTCTGCCGGC
GAACTGCAGAAGGGAAACGAACTGGCCCTGCCCTCCAAATATGTGAACTTCCTGTACCTGGCCAGCCACTATGAG
AAGCTGAAGGGCTCCCCCGAGGATAATGAGCAGAAACAGCTGTTTGTGGAACAGCACAAGCACTACCTGGACGAG

ATCATCGAGCAGATCAGCGAGTTCTCCAAGAGAGTGATCCTGGCCGACGCTAATCTGGACAAAGTGCTGTCCGCC
TACAACAAGCACCGGGATAAGCCCATCAGAGAGCAGGCCAAGAATATCATCCACCTGTTTACCCTGACCAATCTG
GGAGCCCCTGCCGCCTTCAAGTACTTTGACACCACCATCGACCGGAAGAGGTACACCAGCACCAAAGAGGTGCTG
GACGCCACCCTGATCCACCAGAGCATCACCGGCCTGTACGAGACACGGATCGACCTGTCTCAGCTGGGAGGCGAC
AGCCCCAAGAAGAAGAGAAAGGTGGAGGCCAGCTAATAGGACCCAGCTTTCTTGTACAAAGTGGTGACGTAAGCT
AGCAGGATCTTTGTGAAGGAACCTTACTTCTGTGGTGTGACATAATTGGACAAACTACCTACAGAGATTTAAAGC
TCTAAGGTAAATATAAAATTTTTAAGTGTATAATGTGTTAAACTACTGATTCTAATTGTTTGTGTATTTTAGATT
CCAACCTATGGAACTGATGAATGGGAGCAGTGGTGGAATGCCTTTAATGAGGAAAACCTGTTTTGCTCAGAAGAA
ATGCCATCTAGTGATGATGAGGCTACTGCTGACTCTCAACATTCTACTCCTCCAAAAAAGAAGAGAAAGGTAGtt
GACCCCAAGGACTTTCCTTCAGAATTGCTAAGTTTTTTGAGTCATGCTGTGTTTAGTAATAGAACTCTTGCTTGC
TTTGCTATTTACACCACAAAGGAAAAAGCTGCACTGCTATACAAGAAAATTATGGAAAAATATTCTGTAACCTTT
ATAAGTAGGCATAACAGTTATAATCATAACATACTGTTTTTTCTTACTCCACACAGGCATAGAGTGTCTGCTATT
AATAACTATGCTCAAAAATTGTGTACCTTTAGCTTTTTAATTTGTAAAGGGGTTAATAAGGAATATTTGATGTAT
AGTGCCTTGACTAGAGATCATAATCAGCCATACCACATTTGTAGAGGTTTTACTTGCTTTAAAAAACCTCCCACA
CCTCCCCCTGAACCTGAAACATAAAATGAATGCAATTGTTGTTGTTAACTTGTTTATTGCAGCTTATAATGGTTA
CAAATAAAGCAATAGCATCACAAATTTCACAAATAAAGCATTTTTTTCACTGCATTCTAGTTGTGGTTTGTCCAA
ACTCATCAATGTATCTTATCATGTCTGGATCCCGTTTAAACTACGCGTAATTCAAACAGGGTTCTGGCGTCGTTC
TCGTACTGTTTTCCCCAGGCCAGTGCTTTAGCGTTATTGAAAAAGGAAGAGTATGAGTATTCAACATTTCCGTGT
CGCCCTTATTCCCTTTTTTGCGGCATTTTGCCTTCCTGTTTTTGCTCACCCAGAAACGCTGGTGAAAGTAAAAGA
TGCTGAAGATCAGTTGGGTGCACGAGTGGGTTACATCGAACTGGATCTCAACAGCGGTAAGATCCTTGAGAGTTT
TCGCCCCGAAGAACGTTTTCCAATGATGAGCACTTTTAAAGTTCTGCTATGTGGCGCGGTATTATCCCGTATTGA
CGCCGGGCAAGAGCAACTCGGTCGCCGCATACACTATTCTCAGAATGACTTGGTTGAGTACTCACCAGTCACAGA
AAAGCATCTTACGGATGGCATGACAGTAAGAGAATTATGCAGTGCTGCCATAACCATGAGTGATAACACTGCGGC
CAACTTACTTCTGACAACGATCGGAGGACCGAAGGAGCTAACCGCTTTTTTGCACAACATGGGGGATCATGTAAC
TCGCCTTGATCGTTGGGAACCGGAGCTGAATGAAGCCATACCAAACGACGAGCGTGACACCACGATGCCTGTAGC
AATGGCAACAACGTTGCGCAAACTATTAACTGGCGAACTACTTACTCTAGCTTCCCGGCAACAATTAATAGACTG
GATGGAGGCGGATAAAGTTGCAGGACCACTTCTGCGCTCGGCCCTTCCGGCTGGCTGGTTTATTGCTGATAAATC
TGGAGCCGGTGAGCGTGGGTCTCGCGGTATCATTGCAGCACTGGGGCCAGATGGTAAGCCCTCCCGTATCGTAGT
TATCTACACGACGGGGAGTCAGGCAACTATGGATGAACGAAATAGACAGATCGCTGAGATAGGTGCCTCACTGAT
TAAGCATTGGTAACTGTCAGACCAAGTTTACTCATATATACTTTAGATTGATTTAAAACTTCATTTTTAATTTAA
AAGGATCTAGGTGAAGATCCTTTTTGATAATCTCATGACCAAAATCCCTTAACGTGAGTTTTCGTTCCACTGAGC
GTCAGACCCCGTAGAAAAGATCAAAGGATCTTCTTGAGATCCTTTTTTTCTGCGCGTAATCTGCTGCTTGCAAAC
AAAAAAACCACCGCTACCAGCGGTGGTTTGTTTGCCGGATCAAGAGCTACCAACTCTTTTTCCGAAGGTAACTGG
CTTCAGCAGAGCGCAGATACCAAATACTGTTCTTCTAGTGTAGCCGTAGTTAGGCCACCACTTCAAGAACTCTGT
AGCACCGCCTACATACCTCGCTCTGCTAATCCTGTTACCAGTGGCTGCTGCCAGTGGCGATAAGTCGTGTCTTAC
CGGGTTGGACTCAAGACGATAGTTACCGGATAAGGCGCAGCGGTCGGGCTGAACGGGGGGTTCGTGCACACAGCC
CAGCTTGGAGCGAACGACCTACACCGAACTGAGATACCTACAGCGTGAGCTATGAGAAAGCGCCACGCTTCCCGA
AGGGAGAAAGGCGGACAGGTATCCGGTAAGCGGCAGGGTCGGAACAGGAGAGCGCACGAGGGAGCTTCCAGGGGG
AAACGCCTGGTATCTTTATAGTCCTGTCGGGTTTCGCCACCTCTGACTTGAGCGTCGATTTTTGTGATGCTCGTC
AGGGGGGCGGAGCCTATGGAAAAACGCCAGCAACGCGGCCTTTTTACGGTTCCTGGCCTTTTGCTGGCCTTTTGC
TCACATGTTCTTTCCTGCGTTATCCCCTGATTCTGTGGATAACCGTATTACCGCCTTTGAGTGAGCTGATACCGC
TCGCCGCAGCCGAACGACCGAGCGCAGCGAGTCAGTGAGCGAGGAAGCGGAAGA

Figure S1 Nucleotide sequence of pDCC6

U6:96Ab promoter (83..483); sgRNA scaffold (502..577); hsp70Bb promoter (662..1004); 3xFLAG (1105..1173); NLS (1174..1224); hSpCas9 (1225..5325); NLS (5326..5361); SV40 term (5414..6256); ampR (6353..7210)

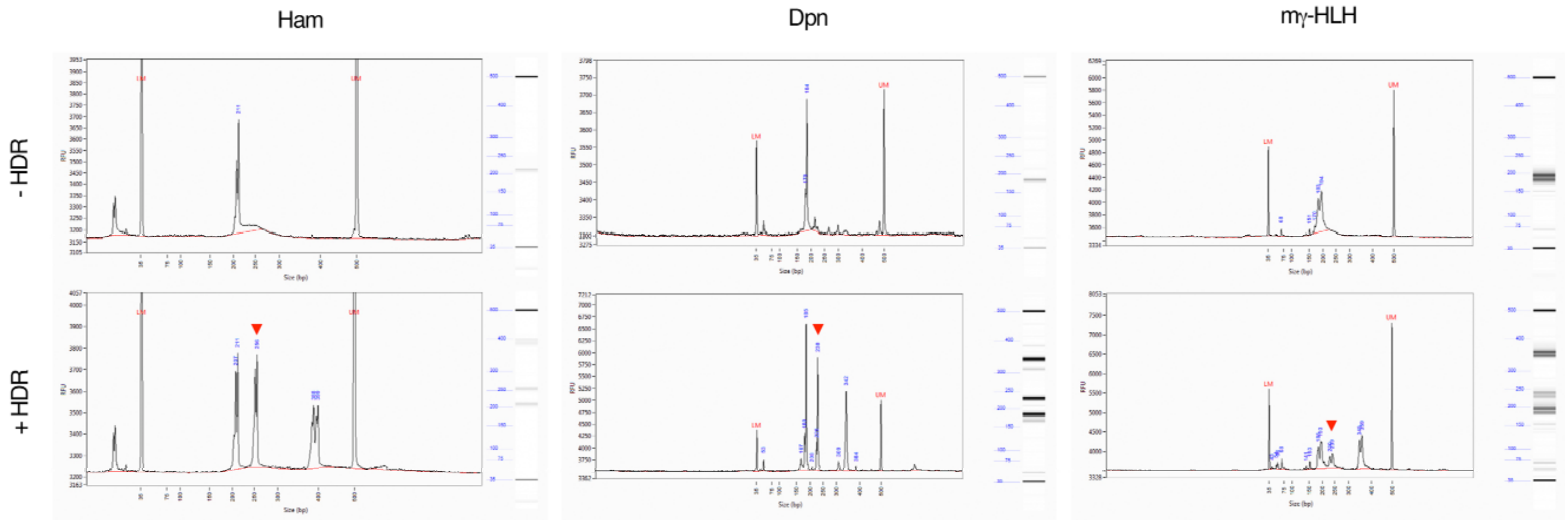

Figure S2 Detection of HDR-mediated V5-tag integration by high-resolution capillary electrophoresis.

PCR samples from heterozygous flies with integrated V5 tags (bottom row) contain an additional larger band/peak of predicted size (red arrowheads) that is not present in untagged chromosomes (top row).
