# Supplementary material for: Efficient CRISPR/Cas9 Plasmids for Rapid and Versatile Genome Editing in Drosophila
Source: G3 (Bethesda). 2014 Sep 17;4(11):2279–82. doi: 10.1534/g3.114.014126 (PMC4232553; doi:10.1534/g3.114.014126)
Supplement: Supporting Information [file supp_g3.114.014126_FileS1.pdf]

File S1

Supplementary Information

gRNA sequences

e	GCCACAATTGTCGATCGTCA	(Port *et al.* 2014)
y	GCGATATAGTTGGAGCCAGC	(Gratz *et al.* 2014)
w	CCGAGAACCTCACCTATGCC	
MED27	CTCCTCGCCGCTCTCCCCAT	
ham	GGCCATTACAATAAGCTCTT	
dpn	CCTGTGCGGTTGATCTACCA	
mγ	GTGAAGTAATCCTATCTACC	

Sequences of recovered indel alleles

e

**ATG**GGTTCGCT**GCCACAATTGTCGATCGTCA**AGGGTCTGCAGCAAGACTT	wt
ATGGGTTCGCT**GCCACAATTGTCGATC-TCA**AGGGTCTGCAGCAAGACTT	- 1
ATGGGTTCGCT**GCCACAATTGTC**--------------TGCAGCAAGACTT	-14
ATGGGTTCGCT**GCCACAATTGTCGA**----**CA**AGGGTCTGCAGCAAGACTT	- 4
ATGGGTTCGCT**GCCACAATTGT**-----**GTCA**AGGGTCTGCAGCAAGACTT	- 5
ATGGGTTCGCT**GCCA**----------------AGGGTCTGCAGCAAGACTT	-16
ATGGGTTCGCT**GCCAC**--------------**A**AGGGTCTGCAGCAAGACTT	-14
ATGGGTTCGCT**GCCACAATTGTCGATC**----------TGCAGCAAGACTT	-10
ATGGGTTCGCT**GCCACAATTGTCGATC**-tgcaaga-CTGCAGCAAGACTT	- 9/+7
ATGGGTTCGCT**GCCACAATTGTCGATCG**a**TCA**AGGGTCTGCAGCAAGACTT	+ 1

MED27

GCCGATGGAACCG**ATGGGGAGAGCGGCGAGGAG**AGCCG	wt
GCCGATGGAACCG**ATG**ctcgaacc**GAGCGGCGAGGAG**AGCCG	-4/+8
GCCGATGGAACCG**ATG-GGAGAGCGGCGAGGAG**AGCCG	-1
GCCGATGGAACCG**ATG**ggagagcggagat**GGGAGAGCGGCGAGGAG**AGCCG	+13

ssODN donor sequences

The homology arms are in upper case, the central V5 sequence in lower case, and the stop codons underlined. The gRNA target sequences are in bold, and are followed by a PAM (NGG). Silent nucleotide changes were introduced in the standard V5 sequence to create a *Hin*dIII restriction site. Donor sequences were synthesized in the same orientation as the gRNA. For all cases presented here, the insertion of the V5 tag destroyed the gRNA target site so that V5-tagged chromosomes would not be recognized and cleaved again.

ham-V5

GCAACATCAGCAACAACAACAGCAGCAGCGGCAACAACAACAACAGCTCCAA**GGCCATTACA**
ATAAGCTCTggtaagcctatacctaaccctcttcttggtctagatagcacg**T**AGGAATCGTA
AGAGATTAGAGAGCTTGCTTTCCTCGTTGTTAAATGATAAACTTACTTTAACTC

dpn-V5

GCAATGAAATAAATTAACAATGTATAAACGTAACATAGAAGTGG**CCTGTGCGGTTGATCTA**c
gtgctatctagaccaagaagagggttaggtataggcttacc**CCA**CGGCCTCCAAGCGGAGCT
CGACTTTTCGATGGCGCCAGCTAGGAATTTCTTGGACGAGGGC

mγ-V5

CTTTCTATGATCTCTCCTTCGTTTTTTTTTTTTTCAGTTGTGGT**GTGAAGTAATCCTATCTA**
cgtgctatctagaccaagaagagggttaggtataggcttacc**CC**AGGGACGCCAGACGTTCT
CCTCGTCCTCGCTGTCGTCCTCCATCTTGGTCACATCGATGGTGGTC
