# Supplementary material for: Efficient CRISPR/Cas9 Plasmids for Rapid and Versatile Genome Editing in Drosophila
Source: G3 (Bethesda). 2014 Sep 17;4(11):2279–82. doi: 10.1534/g3.114.014126 (PMC4232553; doi:10.1534/g3.114.014126)
Supplement: Supporting Information [file supp_g3.114.014126_FigureS2.pdf]

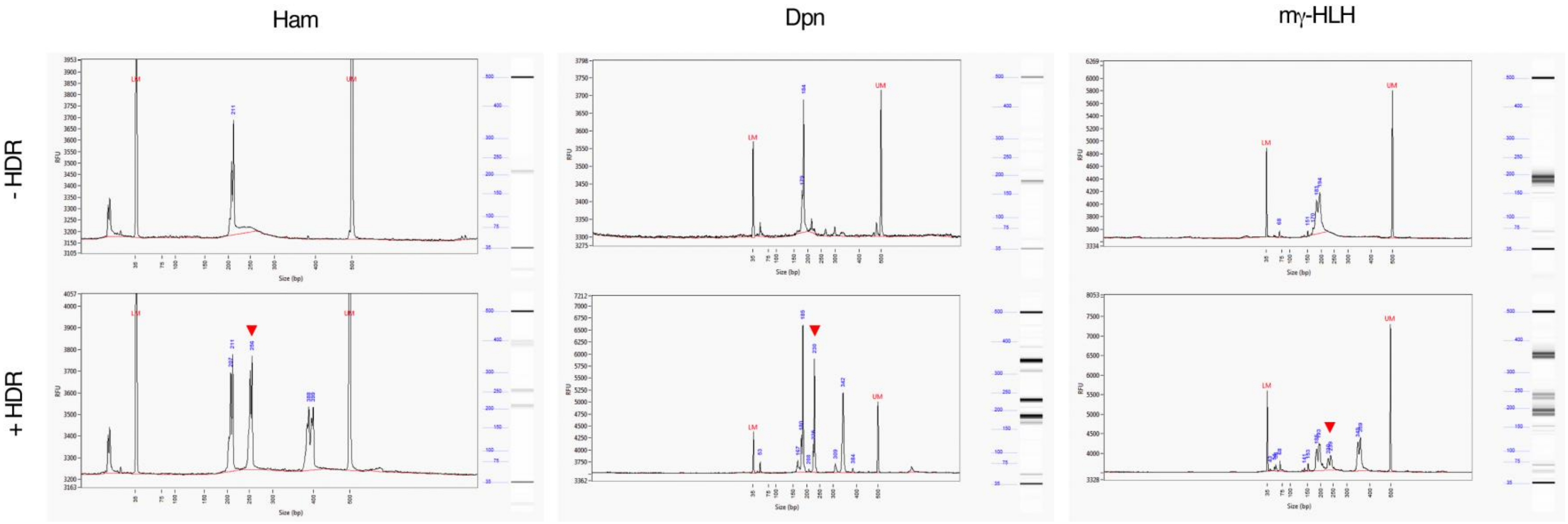

Figure S2 Detection of HDR-mediated V5-tag integration by high-resolution capillary electrophoresis.

PCR samples from heterozygous flies with integrated V5 tags (bottom row) contain an additional larger band/peak of predicted size (red arrowheads) that is not present in untagged chromosomes (top row).
